# Comparison of Depth of Anesthesia in Different Parts of Maxilla When Only Buccal Anesthesia Was Done for Maxillary Teeth Extraction

**DOI:** 10.1155/2011/575874

**Published:** 2011-09-22

**Authors:** Kubilay Isik, Abdullah Kalayci, Ercan Durmus

**Affiliations:** ^1^Department of Oral and Maxillofacial Surgery, Faculty of Dentistry, Baskent University, 06490, Ankara, Turkey; ^2^Baskent Universitesi Konya Uygulama ve Arastirma Merkezi, Konya, Turkey; ^3^Department of Oral and Maxillofacial Surgery, Faculty of Dentistry, Selcuk University, 42079, Konya, Turkey

## Abstract

*Objective*. Recently, some authors reported that maxillary teeth could be extracted without using palatal anesthesia, but they did not clearly specify the extracted teeth. This is important, because apparently the local anesthetic solution infiltrates the maxilla and achieves a sufficient anesthesia in the palatal side. Thus, thickness of the bone may affect the depth of anesthesia. The aim of this study was to compare the depth of anesthesia in different parts of the maxilla when only a buccal infiltration anesthesia was done. *Patients and Method*. The maxilla was divided into anterior, premolar, and molar regions. In each region, 15 teeth were extracted with a single buccal infiltration. The patient marked the pain level on a numerical rating scale. *Results*. Anesthesia depth was sufficient and was not significantly different (*P* > 0.05) among three maxillary regions. *Conclusion*. Except for surgical interventions, all maxillary teeth can be extracted using only a buccal infiltration anesthesia.

## 1. Introduction

The use of palatal anesthesia (PA) is a well-known procedure, and it has been described in detail in textbooks. Since it is a rather painful injection [1], some techniques such as pressure [2], electronic [3], cryogenic [4], or topical anesthesia [5] have been suggested to reduce the patient's discomfort. However, those methods are not universally effective, and PA remains a painful experience for most patients [6].

Recently, it has been claimed that maxillary permanent teeth could be extracted without PA [6, 7]. In those reports, although the indications of extractions were listed, it was not clear which teeth were removed. This is important, because apparently the success of the technique depends on diffusion of any local anesthetic from vestibular side to palatal side [8]. Thus, it can be claimed that while that distance increases, diffusion ability of the local anesthetic to the palatal side will decrease. In other words, a single buccal infiltration anesthesia without PA may be sufficient in anterior maxilla, where the buccopalatal distance is shorter, but it may be not suitable for molar teeth, where buccopalatal distance is longer.

The aim of this study was to investigate if the depth of anesthesia was adequate in all parts of maxilla when only a buccal infiltration anesthesia was done for maxillary permanent teeth extractions.

## 2. Patients and Method

The study was approved by the Ethical Committee of the Baskent University Clinical Researches. Forty-five patients aging between 15 and 76 were included to the study, and one tooth was extracted from each patient. Pediatric patients, the patients who were allergic to Articain and the teeth that needed surgical procedures were excluded.

The maxilla was divided into three regions, and 15 teeth were extracted from each region as follows:

- anterior: central, lateral, and canine teeth,
- premolar: first and second premolars,
- molar: first and second molars.

**Figure 1:**
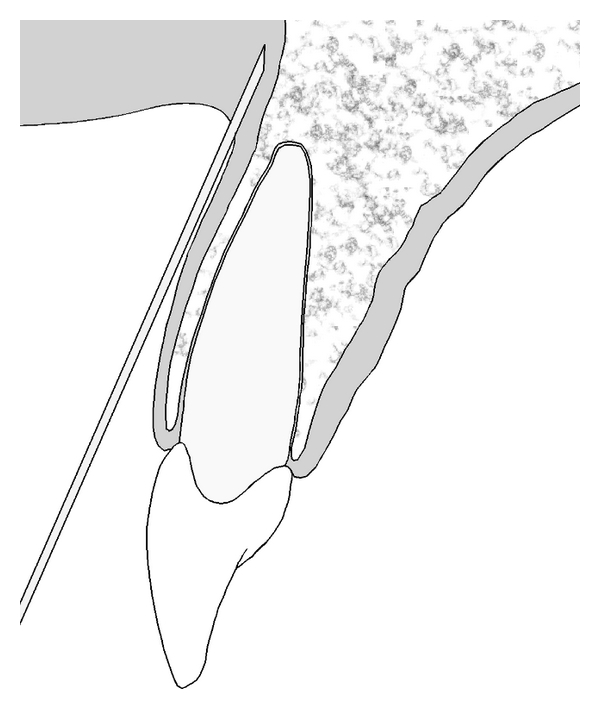
Technique of buccal infiltration anesthesia.

2 mL of local anesthetic solution containing 80 mg Articain HCl and 0.012 mg epinephrine was used (Ultracain D-S, Sanofi-Aventis, Istanbul, Turkey). 1.7 mL of the solution was injected according to conventional methods [9] (Figure 1). The remaining 0.3 mL of the solution was left to use for PA if the patient would have a pain during the extraction. All teeth were anesthetized with a single buccal injection. Infraorbital anesthesia or posterior alveolar nerve blocks were not employed.

After waiting five minutes, as suggested by Uckan et al. [7], the numbness of the palatal mucosa was gently checked with a dental probe and the patient asked if it was painful. The patient was told to warn us if he would have felt a moderate or severe pain during the extraction. Then, the tooth was slowly extracted in usual way. The patient was also asked to mark the extraction pain on an 11-point numerical rating scale (between 0 and 10) anchored with the expressions “no pain” and “the worst pain imaginable” on its ends.

The pain scores were statistically analyzed with the Kruskal Wallis test by using a commercial software (SigmaStat v3.5, SysStat Software, Richmond, Calif, USA). Statistical significance was accepted at 95% confidence level.

## 3. Results

All patients tolerated the extractions well, and none of them reported severe pain. All patients verbally described the procedure “totally painless” or “a very slight discomfort” (Table 1). Statistical analysis revealed that there were no significant difference among three maxillary regions (*P* > 0.05). The palatal mucosa probing was slightly or moderately painful in all patients. Since the aim of this study was to evaluate only the extraction procedure, no statistical analysis was performed for palatal mucosa probing results.

## 4. Discussion

Palatal injection for permanent maxillary tooth removal is poorly tolerated by the patients, and it is one of the most painful procedures in dentistry [10, 11]. Piercing the mucosa is painful to a degree, but the main source of the pain is displacement of the mucoperiosteum [12]. To overcome this, many techniques have been suggested [2–5], but none of them is universally effective, and some of them even require specific equipment [13].

Recently some authors reported that maxillary erupted the third molars, and other permanent teeth could be extracted by using only buccal infiltration anesthesia [6, 7, 14]. There are three opinions explaining the efficiency of the technique. First, it has been advocated that the anesthetic requirement for tooth extraction is not as high as that required for routine conservative dental treatment [15]. Second, it has been claimed that Articaine diffuses more readily through soft and hard tissues than other local anesthetics [7]. Finally, it has been suggested that the porous nature of the maxilla facilitates the diffusion of any local anesthetic [8].

All of those opinions may be true and valid, but infiltration of the local anesthetic solution to the palatal side should be the most determinative factor. That also makes the distance between the buccal and palatal side of the maxillary alveolus important. Because it is obvious that diffusion of the solution to the palatal side will not be the same in a thicker alveolus. The authors who performed the technique and reported successful results [6, 7] listed the indications for the extractions (wisdom teeth, orthodontic teeth, fractured teeth, profound caries, periodontitis, etc.) and even reported a success rate according to indications (orthodontic treatment > periodontitis > prophylactic extraction > apical lesion > profound caries) [7]. However, in those reports, it was not clear which teeth were extracted. The goal of this study was to classify the maxillary teeth according to buccopalatal alveolar ridge thickness and to find if the technique is effective in all parts of the maxillary alveolus.

While, according to classical knowledge, 2-3 minutes will be sufficient in buccal infiltration anesthesia [9], a prolonged delay is necessary in this technique to allow diffusion of the solution to the palatal side. All of the patients described the extraction “completely painless” or a “very slight, faint pain,” and there was no significant difference among three maxillary regions. However, it should be noted that all of the patients reported a considerable pain when probing the palatinal mucosa. Therefore, extraction of maxillary teeth without using PA is not suitable for surgical extractions or for the procedures in which the palatal mucosa will be manipulated by elevators or will be sutured.

The technique should be effective in pediatric patients, whose alveolar ridges are narrower than adults, as well. However, pediatric patients were not included to the study because they might not express the pain correctly. Upper third molars were also not included to the study, but it has already been demonstrated that the technique is successful for erupted upper third molars [14].

## 5. Conclusion

After an enough delay, it is possible to extract the maxillary teeth without PA and the technique is effective for all maxillary teeth. However, PA, is a must for the teeth requiring surgical procedures.

## Figures and Tables

**Table 1:** Pain scores that were obtained during teeth extractions from three different maxillary regions.

Anterior	Premolar	Molar
1	1	2
1	1	1
0	0	1
0	0	0
0	0	0
0	0	0
0	0	0
0	0	0
0	0	0
0	0	0
0	0	0
0	0	0
0	0	0
0	0	0
0	0	0
